# Recent introgression between Taiga Bean Goose and Tundra Bean Goose results in a largely homogeneous landscape of genetic differentiation

**DOI:** 10.1038/s41437-020-0322-z

**Published:** 2020-05-26

**Authors:** Jente Ottenburghs, Johanna Honka, Gerard J. D. M. Müskens, Hans Ellegren

**Affiliations:** 1 Department of Evolutionary Biology, University of Uppsala, Uppsala, Sweden; 2 Department of Ecology and Genetics, University of Oulu, PO Box 3000, FI-90014 Oulu, Finland; 3 Team Animal Ecology, Wageningen Environmental Research, Wageningen University & Research, Droevendaalsesteeg 3-3A, 6708 PB Wageningen, The Netherlands

**Keywords:** Speciation, Evolutionary genetics, Population genetics, Evolutionary biology

## Abstract

Several studies have uncovered a highly heterogeneous landscape of genetic differentiation across the genomes of closely related species. Specifically, genetic differentiation is often concentrated in particular genomic regions (“islands of differentiation”) that might contain barrier loci contributing to reproductive isolation, whereas the rest of the genome is homogenized by introgression. Alternatively, linked selection can produce differentiation islands in allopatry without introgression. We explored the influence of introgression on the landscape of genetic differentiation in two hybridizing goose taxa: the Taiga Bean Goose (*Anser fabalis*) and the Tundra Bean Goose (*A. serrirostris*). We re-sequenced the whole genomes of 18 individuals (9 of each taxon) and, using a combination of population genomic summary statistics and demographic modeling, we reconstructed the evolutionary history of these birds. Next, we quantified the impact of introgression on the build-up and maintenance of genetic differentiation. We found evidence for a scenario of allopatric divergence (about 2.5 million years ago) followed by recent secondary contact (about 60,000 years ago). Subsequent introgression events led to high levels of gene flow, mainly from the Tundra Bean Goose into the Taiga Bean Goose. This scenario resulted in a largely undifferentiated genomic landscape (genome-wide *F*_ST_ = 0.033) with a few notable differentiation peaks that were scattered across chromosomes. The summary statistics indicated that some peaks might contain barrier loci while others arose in allopatry through linked selection. Finally, based on the low genetic differentiation, considerable morphological variation and incomplete reproductive isolation, we argue that the Taiga and the Tundra Bean Goose should be treated as subspecies.

## Introduction

It is increasingly appreciated that interspecific gene flow, or introgression, is a common phenomenon. Numerous species have exchanged genetic material with other species through introgressive hybridization (Barlow et al. 2018; Palkopoulou et al. 2018; Árnason et al. 2018; Wu et al. 2018; Gopalakrishnan et al. 2018), including our own species, *Homo sapiens* (Patterson et al. 2012; Vernot et al. 2016; Villanea and Schraiber 2018). This widespread genetic exchange has changed our views on the evolutionary process and the nature of species (Mallet et al. 2016; Shapiro et al. 2016; Roux et al. 2016).

A number of studies have revealed a highly heterogeneous landscape of genetic differentiation across the genomes of closely related species (Turner et al. 2005; Nadeau et al. 2012; Ellegren et al. 2012; Renaut et al. 2013). Genetic differentiation (measured for example by *F*_ST_, the fixation index) between species pairs is often concentrated in a few genomic regions, the so-called islands of differentiation (Wolf and Ellegren 2017). This finding led to the formulation of a verbal model in which such islands diverge over time (i.e. higher absolute divergence, *d*_*XY*_) because they contain loci involved in reproductive isolation (and hence originally referred to as “genomic islands of speciation”, Turner et al. 2005), whereas the rest of the genome is homogenized by interspecific gene flow (Wu 2001; Turner et al. 2005; Feder et al. 2012). This leads to small genomic regions of high divergence against a background of low divergence.

Rigorous tests of islands of differentiation have revealed that reduced diversity due to linked selection can also lead to heterogeneous genomic landscapes (Cruickshank and Hahn 2014; Wolf and Ellegren 2017). This is thought to arise from two processes: genetic hitchhiking or background selection (Cutter and Payseur 2013; Burri 2017; Rettelbach et al. 2019; Stankowski et al. 2019; Buffalo and Coop 2019). Genetic hitchhiking refers to the situation in which positive selection on a variant results in selection for the genetic region in which this advantageous variant occurs. As the advantageous variant goes toward fixation, loci linked to this variant hitchhike along and increase in frequency (Smith and Haigh 1974). Background selection involves purifying selection against recurring deleterious mutations (Charlesworth 1994). This process also reduces diversity at linked sites. Genomic regions with high levels of recombination are expected to experience less linked selection because recombination uncouples loci from the advantageous or deleterious variant under selection (Hudson and Kaplan 1995; Nordborg et al. 1996). These processes–genetic hitchhiking and background selection—can produce islands of differentiation in allopatry in the absence of gene flow.

The True Geese (genera *Anser* and *Branta*) are an excellent system to explore the consequences of introgressive hybridization on a genomic level (Ottenburghs et al. 2016a). Previous work has uncovered introgression between several goose species (Ottenburghs et al. 2016b, 2017a), but it remains to be determined when these introgression events occurred and how these species remain distinct in the face of gene flow. In this study, we focus on two Bean Goose taxa: the Taiga Bean Goose (*Anser fabalis*) and the Tundra Bean Goose (*A. serrirostris*). These taxa belong to the Bean Goose complex (which also includes the Pink-footed Goose, *A. brachyrhynchus*) and have been considered conspecific based on morphology (Delacour 1951; Sangster and Oreel 1996; Mooij and Zöckler 1999) and mitochondrial DNA (Ruokonen et al. 2008). Genomic analyses have indicated that divergence within the Bean Goose complex occurred ~2 million years ago (Ottenburghs et al. 2016b). Moreover, ecological evidence suggests that the Taiga and the Tundra Bean Goose might be distinct species since they use different breeding grounds (Burgers et al. 1991) and show differences in behavior and vocalizations (Sangster and Oreel 1996). Also, slight differences in morphology exist between the taxa in body size, shape, plumage patterns and in beak morphology and coloration: the Taiga Bean Goose has a longer beak with a broad orange marking whereas the Tundra Bean Goose has a shorter beak with a reduced orange band on the bill. However, a recent study showed that only two measurements out of total of 17 distinguished the Taiga and the Tundra Bean Goose from each other (de Jong 2019), thus considerable interspecific overlap exists. Hybrids between taxa of the Bean Goose complex have been reported (Ottenburghs et al. 2016a; Honka et al. 2017), mainly based on genetic tests because hybrids are difficult to identify due to morphological similarities with both parental species (Randler 2004). Moreover, most hybrids were reported during migration and on the wintering grounds, so it is currently not possible to pinpoint a putative hybrid zone on their breeding areas. Whether the hybrids are fertile and backcross with the parental species—and thus resulting in introgression–remains to be investigated.

In this study, we explore the evolutionary history of the Taiga and the Tundra Bean Goose using whole-genome re-sequencing data (on average 37× coverage with paired-end sequencing). We investigate (1) how genetic differentiation is distributed across the genome and (2) how the timing of introgression influences the structure of the genomic landscape of differentiation. We address these questions through a combination of population genomic summary statistics, including relative divergence (*F*_ST_), absolute divergence (*d*_*XY*_), nucleotide diversity (*π*) and Tajima’s *D*. We also apply demographic modeling. Finally, we assess the taxonomic status of the Taiga and the Tundra Bean Goose, which has been heavily debated, by combining the genetic results with morphological and ecological information. Moreover, the Taiga Bean Goose is declining: population numbers have halved since the 1990s, but the Taiga Bean Goose is still being hunted. Current population size estimates are 53,000–57,000 individuals for the Taiga Bean Goose and 600,000 individuals for the Tundra Bean Goose (Fox and Leafloor 2018). Thus, verifying the taxonomical position of the Taiga and Tundra Bean Goose is of utmost importance for the correct management of the taxa.

## Material and methods

### Sequencing and quality assessment

**Fig. 1:**
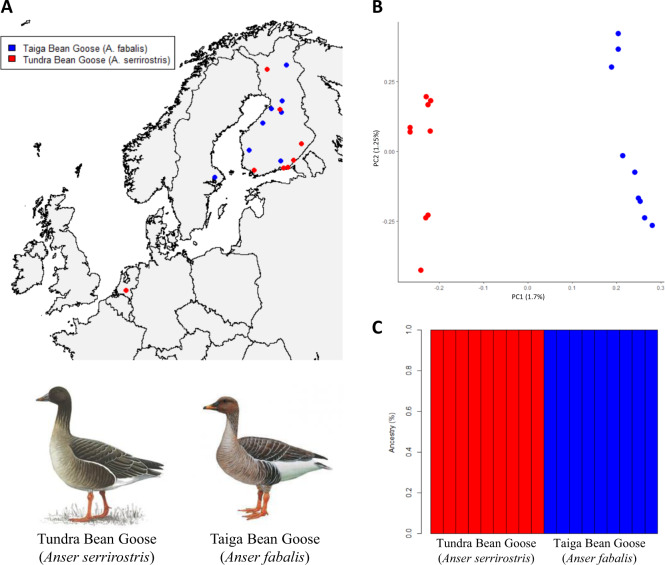
Population genetic structure of Taiga and Tundra Bean Goose. **a** Map of sampling locations of migrating Taiga and Tundra Bean Goose. **b** Principal component analysis (PCA) and **c** *ADMIXTURE-*analyses show clear genetic differentiation between Taiga and Tundra Bean Goose. Drawings used with permission of the Handbook of the Birds of the World (del Hoyo et al. 2018).

We collected blood and tissue samples for the Taiga Bean Goose (*A. fabalis*, *n* = 9) and the Tundra Bean Goose (*A. serrirostris*, *n* = 9), migrating within Europe (Fig. 1a, Supplementary Table S1). Due to elusive nature of the species, especially during the breeding time, and remote breeding sites (mires and tundra-like habitats), the samples were collected from legally hunted geese during their migration. The tissue samples were collected in years 2010–2013 and stored frozen in absolute ethanol. Genomic DNA was isolated from the blood and tissue samples using the Qiagen Gentra kit (Qiagen Inc.). Quality and quantity of the DNA was measured using the Qubit (Invitrogen, Life Technologies).

Sequencing libraries were prepared from 100 ng DNA using the TruSeq Nano DNA sample preparation kit (cat# FC-121-4001/4002, Illumina Inc.), targeting an insert size of 350 bp and a target coverage of 30×. Whole-genome paired-end sequencing (150 bp) was performed on an Illumina HiSeqX following standard procedures. Sequencing reads were mapped to the reference genome of a closely related goose species with the highest quality, namely Swan Goose (*Anser cygnoides*) genome version 1.0 (Gao et al. 2016), using Burrows–Wheeler Aligner (BWA) version 0.7.17 (Li and Durbin 2009). The resulting BAM-files were sorted with Samtools version 1.6 (Li et al. 2009) and duplicates were marked with Picard version 2.10.3 (http://broadinstitute.github.io/picard/). Next, local realignment was performed using GATK version 3.7 (McKenna et al. 2010).

For each individual, a first round of variant calling was performed with GATK HaplotypeCaller. The resulting list of variants was filtered on mapping quality (MQRankSum < 0.22) and read depth (DP > 10). The variants passing these filters were then used as a reference set for base quality score recalibration (BQSR) following a bootstrapping approach in GATK (following Kardos et al. 2018). Next, we applied a hard filter in line with the GATK best practices pipeline (Van der Auwera et al. 2013), applying the following filtering criteria: QD < 2.0 | | FS > 60.0 | | MQ < 40.0 | | MQRankSum < −12.5 | | ReadPosRankSum < −8.0. The final dataset contained 13,890,330 SNPs. Different filtering steps were applied in the consequent analyses.

### Population structure and differentiation

Using VCFtools version 0.1.15 (Danecek et al. 2011), we removed loci for which the *p*-value was smaller than 0.01 in a test for excess of heterozygotes relative to Hardy–Weinberg genotype proportions. Moreover, we retained only loci with a minor allele frequency ≥ 0.05. Finally, the SNPs were filtered on linkage disequilibrium along windows of 50 markers with a *R*^2^-threshold of 0.5. The resulting dataset of 6,221,883 SNPs provided the input for the principal component analysis (PCA) using the pca-function in Plink version 1.07 (Purcell et al. 2007). These analyses were repeated with different settings for the Hardy–Weinberg test and linkage disequilibrium to assess the robustness of the patterns.

The same dataset of 6,221,883 SNPs was used to assess the ancestry composition of each individual in *ADMIXTURE* version 1.3.0 (Alexander et al. 2009). All SNPs were formatted for the *ADMIXTURE-*analyses (i.e. converted to BED-format) using Plink version 1.07 (Purcell et al. 2007). We ran analyses with the number of clusters set from *K* = 1 to 4, and performed 10-fold cross-validation to assess the optimal number of clusters. The final admixture proportions per individual (*Q*-estimates representing the log-likelihood of cluster assignment) were visualized with R version 3.5.0 (R Core Team 2018).

The filtered dataset of 13,890,330 SNPs was used to construct the genomic landscape of differentiation. Summary statistics were calculated across non-overlapping windows of 200,000 nucleotides (200 kb). To assess the genome-wide heterogeneity in genetic differentiation, we calculated relative divergence (*F*_ST_). However, this statistic is a relative measure of differentiation that is dependent on the underlying genetic diversity within the population (Ottenburghs et al. 2017b; Wolf and Ellegren 2017). Therefore, we also estimated absolute divergence (*d*_*XY*_) and nucleotide diversity (*π*) to rule out any effects of local reductions in genetic diversity on patterns of genetic differentiation. Finally, to infer whether these regions of reduced genetic diversity are the result of (linked) selection, we calculated Tajima’s *D*. Negative values of this statistic suggest purifying selection or population expansion (Tajima 1989). Moreover, divergent selection is expected result in higher absolute divergence (*d*_*XY*_) and lower nucleotide diversity (*π*) in particular genomic regions. Hence, we correlated *F*_ST_ with *d*_*XY*_ and *π*. Relative divergence (*F*_ST_) and Tajima’s *D* were calculated using VCFtools version 0.1.15 (Danecek et al. 2011), whereas absolute divergence (*d*_*XY*_) and nucleotide diversity (*π*) were calculated with the popgenWindows.py script from Martin et al. (2015) which is available here: https://github.com/simonhmartin/genomics_general. The analyses were repeated for different window sizes (10, 20, 50 and 100 kb) to rule out any effects of window size.

Because the Swan Goose genome has not been assembled on a chromosome level, we aligned scaffolds to the highest quality bird genome currently available, namely the Chicken (*Gallus gallus*) genome assembly Galgal6 (Hillier et al. 2004), with LASTZ version 1.04.00 (Harris 2007). The scaffolds were ordered and orientated based on the coordinates from the Chicken genome and consequently merged into pseudo-chromosomes. The resulting alignment was visualized with R version 3.5.0 (R Core Team 2018) using the package ggplot2 (Wickham 2016).

### Demographic analyses

Demographic inference was performed using the software package DADI (Gutenkunst et al. 2009). Because demographic analyses can be biased by selection (Ragsdale et al. 2018), we only used non-coding loci (5,397,934 SNPs). These loci were selected using snpEff version 4.3T (Cingolani et al. 2012), which annotates SNPs into several functional classes, such as protein-coding, intronic, and intergenic regions. Due to the lack of an outgroup to establish the ancestral state for each SNP, we used a folded frequency spectrum. We tested several demographic models with increasing complexity to estimate the timing of gene flow between the Taiga and the Tundra Bean Goose, ranging from strict isolation to secondary contact with asymmetrical gene flow. For each scenario, ten simulations were run with different starting values to ensure proper exploration of the likelihood landscape. After convergence of parameters, the simulation with the highest likelihood was retained. The final set of parameters was converted into absolute time and population size estimates using a mutation rate of 1 × 10^−9^ per nucleotide per generation (Pujolar et al. 2018) and a generation time of two years (Ottenburghs et al. 2017a). Confidence intervals for parameters were generated using a bootstrap approach (10 iterations) in which 1 million SNPs were randomly selected and a demographic model was tested with DADI using the parameter values from the most likely model as a starting point.

## Results

### Sequencing and quality assessment

We re-sequenced the genomes of nine Taiga Bean Geese and nine Tundra Bean Geese. All 18 samples were mapped to the Swan Goose genome (Supplementary Table S2), with an average mapping percentage of 92.6% (range: 83.7–97.6) and an average sequencing depth of 37× (range: 31–44). SNP calling, following the GATK best practices guidelines (Material and methods), resulted in a final dataset of 13,890,330 SNPs.

### Population structure and differentiation

**Fig. 2:**
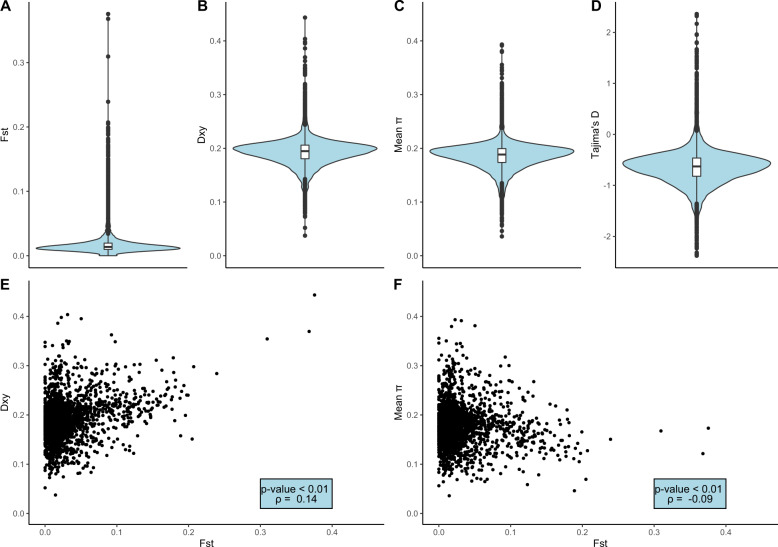
Distribution of summary statistics. **a** Relative divergence (*F*_ST_), **b** absolute divergence (*d*_*XY*_), **c** nucleotide diversity (*π*) and **d** Tajima’s *D*. Correlations between **e** *F*_ST_ and *d*_*XY*_ and between **f** *F*_ST_ and nucleotide diversity.

The PCAs indicated that the Taiga and the Tundra Bean Goose can be separated using genomic data. The first principal component discriminated between the two taxa and the second principal component indicated some intraspecific population structure within both taxa (Fig. 1b). This intraspecific population structure might relate to the distribution of breeding areas, but unfortunately we do not have information about sites of origin because the birds were sampled during migration. The principal components explained little genetic variance, suggesting that only a subset of genetic loci drive the genetic differences between the taxa. However, the PCA-patterns were robust to different filtering settings (Supplementary Fig. S1). In contrast, the individual ancestries estimated by *ADMIXTURE* pointed to one population (*K* = 1 had the lowest CV-error, Supplementary Fig. S2) although the analyses with *K* = 2 confidently discriminated between two genetically distinct populations under particular filtering criteria (Fig. 1c). Moreover, relaxing the thresholds for linkage disequilibrium and minor allele frequency in filtering the SNPs highlighted a more admixed pattern (Supplementary Fig. S1), suggesting that a large proportion of genetic variation is shared between the taxa. This observation is confirmed by the genomic window analyses which show that genetic divergence was concentrated in a small number of differentiated loci. The majority of genomic windows showed low levels of *F*_ST_ (genome-wide *F*_ST_ = 0.033, Fig. 2a) and intermediate values of *d*_*XY*_ (Fig. 2b) and *π* (Fig. 2c). Most genomic windows showed a negative value for Tajima’s *D* (Fig. 2d), which can be due to purifying selection or population expansion (Tajima 1989). High *F*_ST_-windows were characterized by slightly higher levels of absolute divergence (*d*_*XY*_, Spearman correlation, *ρ* = 0.14, *p* < 0.01, Fig. 2e) and lower levels of nucleotide diversity (Spearman correlation, *ρ* = −0.16, *p* < 0.01, Fig. 2f). These results were robust against different window sizes (Supplementary Table S3).

**Fig. 3:**
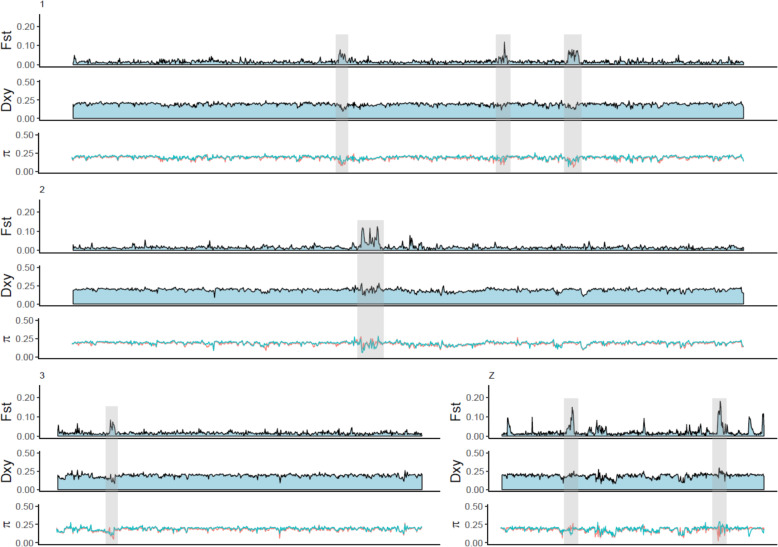
The genomic landscape of Taiga and Tundra Bean Goose, with sequences aligned to chicken chromosomes, for relative divergence (*F*_ST_), absolute divergence (*d*_*XY*_) and nucleotide diversity (*π*). The colors in the nucleotide diversity tracks correspond to the Taiga (blue) and the Tundra Bean Goose (red). Only the first three chromosomes and the Z-chromosome are depicted, a complete picture is provided in Supplementary Fig. S3. The highlighted areas (gray boxes) show examples of differentiated islands.

The results from Fig. 2 were visualized in the genomic landscape of differentiation (Fig. 3, Supplementary Fig. S2). The *F*_ST_-landscape was largely flat with a few notable peaks that were scattered across chromosomes (82 *F*_ST_-windows above 0.25). Peaks in *F*_ST_ were often accompanied by lower levels of *d*_*XY*_ and a drop in nucleotide diversity in one or both taxa (e.g., highlighted regions on chromosomes 1, 2 and 3 in Fig. 3). However, in some cases, a peak in *F*_ST_ corresponded to an increase in *d*_*XY*_ (e.g., highlighted regions on the Z-chromosome in Fig. 3). Although there were a few notable *F*_ST_*-*peaks on the Z-chromosome (Fig. 3), the mean *F*_ST_ between windows on the autosomes and the Z-chromosome was not significantly different (two-sample *t*-test, *t* = 0.49, *p* = 0.62).

### Demographic analyses

**Fig. 4:**
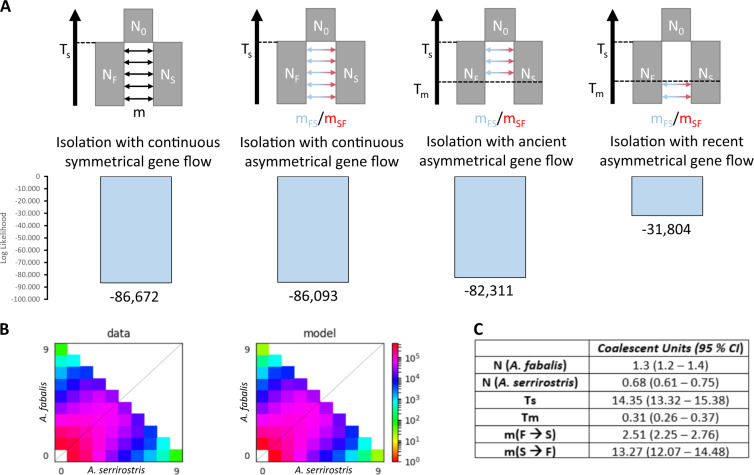
Demographic analyses point to recent asymmetrical gene flow. **a** Four different demographic scenarios and their likelihood scores that were tested with DADI. The most likely model concerns isolation with recent asymmetrical gene flow (log-likelihood=−31,804). **b** Comparison between the actual data and the simulated model and **c** the parameters (in 2N-units) estimated in the most likely demographic model.

Demographic modeling indicated that a model of strict isolation was highly unlikely (log-likelihood = −472,672). The inclusion of gene flow markedly improved the likelihood estimation, as exemplified by the log-likelihood of a model with continuous, symmetrical gene flow was −86,672. Exploration of more sophisticated models with asymmetrical gene flow indicated that the most likely model (log-likelihood = −31,804) entails a scenario of secondary contact with gene flow mainly from the Tundra into the Taiga Bean Goose (Fig. 4a, b, Supplementary Table S4). Including population expansions for one of both taxa, did not improve the likelihood scores (Supplementary Fig. S4, Supplementary Table S4). Transforming the coalescent units (Fig. 4c, Supplementary Table S5) into absolute time showed that the taxa diverged ~2.66 million years ago (95% CI: 2.47–2.81 million years) and that secondary contact occurred around 58,285 years ago (95% CI: 48,658–67,918 years). Effective population sizes after the initial split were 102,508 (95% CI: 110,954–130,061) and 62,855 (95% CI: 56,102–69,608) for the Taiga Bean Goose and the Tundra Bean Goose, respectively.

## Discussion

### The evolutionary history of the Bean Geese

Our genomic analyses indicated that the Taiga Bean Goose and the Tundra Bean Goose can be genetically separated despite overlapping values in most morphological traits and gene flow (Fig. 1). Moreover, the demographic modeling revealed that the taxa diverged ca. 2.66 million years ago (Fig. 4), in line with previous estimates (Ruokonen et al. 2000; Ottenburghs et al. 2016b). This divergence time coincides with a fast global cooling trend that resulted in a circumpolar tundra belt and expansion of temperate grasslands (Zachos et al. 2001), the ideal habitats for geese to thrive (Owen 1980). After a period of allopatry, the Taiga and the Tundra Bean Goose established secondary contact about 60,000 years ago which culminated in bidirectional gene flow, though mostly from the Tundra into the Taiga Bean Goose.

The period of introgression occurred during the Weichselian Glaciation (between 75,000 and 11,000 years ago) when a cooling trend introduced tundra vegetation in the Northern hemisphere (Mangerud et al. 2011; Otvos 2015). During this period, the geese probably resided in different refugia: the Taiga Bean Goose was driven to southwestern Europe (specifically Spain) whereas the Tundra Bean Goose occurred on the tundra in western Siberia (Ploeger 1968). The warm interstadials during the Weichselian cooling period might have brought these populations in secondary contact. On the basis of the current distributions, we can assume that the Taiga Bean Goose moved northwards into the range of the Tundra Bean Goose. Initially, the moving Taiga Bean Goose might have been outnumbered by the Tundra Bean Goose in certain areas, leading to hybridization. As the range shift proceeded, the Tundra Bean Goose and previously produced hybrids were probably incorporated into the Taiga Bean Goose population, thereby overturning the numerical imbalance. Consequently, hybrids might have had a higher chance of backcrossing with the Taiga Bean Goose, resulting in the observed pattern of asymmetric gene flow from Tundra into Taiga Bean Goose (Currat et al. 2008). These findings support the widespread occurrence of introgressive hybridization between bird species in general (Rheindt and Edwards 2011; Ottenburghs et al. 2017b), and geese in particular (Ottenburghs et al. 2017a).

### Islands of differentiation

Although it is possible to discriminate between the Taiga and the Tundra Bean Goose using genetic data (Fig. 1), it does not automatically follow that the taxa are genetically distinct. Indeed, PCAs tend to overemphasize differences (Björklund 2019) and *ADMIXTURE*-analyses are sensitive to filtering criteria applied to the SNPs (Lawson et al. 2018). These biases were also apparent in our analyses. Regardless of the filtering thresholds, PCAs clearly discriminated between both taxa. In the *ADMIXTURE*-analyses, on the other hand, more stringent filtering criteria uncovered varying levels of shared ancestry between the Taiga and the Tundra Bean Goose (Supplementary Fig. S2). These findings indicate the potential issues of solely relying on PCAs and genetic ancestry analyses when assessing the genetic make-up of populations. Therefore, it is important to investigate genetic patterns in more detail, for example by exploring the genomic landscape of differentiation.

In line with the *ADMIXTURE*-analyses, the genetic divergence between the taxa seems to be driven by few genomic regions that are scattered throughout the genome, so-called islands of differentiation (Figs. 2 and 3). This pattern has been observed in other bird species, such as crows (Poelstra et al. 2014), woodpeckers (Grossen et al. 2016), warblers (Toews et al. 2016; Irwin et al. 2018), flycatchers (Ellegren et al. 2012; Burri et al. 2015), thrushes (Ruegg et al. 2014; Delmore et al. 2015), stonechats (Van Doren et al. 2017), and nightingales (Mořkovský et al. 2018). In line with previous studies, we found no significant difference in the level of genetic differentiation between islands on autosomes and on the Z-chromosome (e.g., Ellegren et al. 2012; Bay and Ruegg 2017; Mořkovský et al. 2018). Some patterns suggest that some of the islands of differentiation uncovered in this study might contribute to reproductive isolation, whereas the remainder of the genome can freely flow between the species. The positive correlation between *F*_ST_ and *d*_*XY*_ indicated increased genetic divergence in particular genomic regions, whereas the rest of the genome showed divergence levels close to the genome-wide average (Fig. 2e). In addition, the demographic model uncovered high levels of recent gene flow between the Taiga and the Tundra Bean Goose (Fig. 4). The islands of differentiation contained some interesting candidate genes, such as *KCNU1*, which is involved in spermatogenesis and might thus play a role in prezygotic post-mating isolation (Buffone et al. 2012). However, more detailed analyses are needed to validate these candidate genes (see Supplementary Table S6 for a list of candidate genes).

Several patterns indicated that the genomic landscape of the Bean Geese was at least in part shaped by linked selection. The negative correlation between *F*_ST_ and nucleotide diversity suggests that selection reduced genetic diversity in certain genomic regions (Fig. 2f). Negative values of Tajima’s *D* across the majority of genomic windows (Fig. 2d) point to purifying selection or population expansion (Tajima 1989). Also, some of the differentiation islands did not show elevated *d*_*XY*_ values, indicating linked selection (Fig. 3). Few of the high *F*_ST_ islands were accompanied by a decrease in absolute divergence *d*_XY_ (Fig. 3). Instead of extant linked selection that does not cause a drop in *d*_XY_, this result can be explained by recurrent selection (i.e. selection in a common ancestor and in the daughter species, Cruickshank and Hahn 2014; Irwin et al. 2018). Clearly, more detailed analyses are needed to determine the relative contributions of reproductive isolation and linked selection in shaping the genomic landscape of the Bean Geese. Such analyses include quantifying the relationship between levels of diversity and local recombination rate, and comparing the genomic landscapes of related goose species (Burri et al. 2015; Ravinet et al. 2017; Stankowski et al. 2019). The islands of differentiation may evolve at the same genomic regions at independent lineages even across broad taxonomical range due to linked selection at conserved genetic elements such as areas of low recombination (Burri et al. 2015; Dutoit et al. 2017; Delmore et al. 2018).

### Taxonomic recommendations

The degree and character of genomic differences between the Taiga and the Tundra Bean Goose raise the question whether they should be considered separate species. Specifically, do a few differentiated regions in the genome provide enough evidence to consider them as distinct species? As a single criterion, genomic differentiation might be considered too low to justify a species rank. But, in combination with other species criteria, such as morphology, behavior and ecology, genomics could provide an extra line of evidence in species classification (Ottenburghs 2019). Indeed, avian taxonomy has become more pluralistic (Sangster 2018), combining different species criteria to justify taxonomic decisions (Alström et al. 2008; Gohli et al. 2015; Oswald et al. 2016).

Furthermore, linking islands of differentiation to other species criteria, such as morphology or reproductive isolation, can strengthen a taxonomic decision. This is nicely illustrated by the genomic analyses of Hooded Crow (*Corvus cornix*) and Carrion Crow (*C. corone*), which uncovered a single differentiated genomic region that harbored several genes involved in pigmentation and visual perception (Poelstra et al. 2014). These genetic variants have been shown to underlie the different plumage patterns (black or gray-coated) in these species (Wu et al. 2019; Knief et al. 2019). In addition, several behavioral studies uncovered assortative mating according to plumage phenotypes (Saino and Villa 1992; Risch and Andersen 1998; Haas et al. 2010). Such detailed investigations have not been performed for the Bean Goose complex. The genomic islands of differentiation uncovered in this study might be associated with morphological and behavioral differences between the Taiga and the Tundra Bean Goose, but this remains to be determined by denser sampling across the range of these taxa and experimental work on their social behavior.

On the basis of the evidence from different species criteria (e.g., genetic differentiation, reproductive isolation and morphology), one can thus assess the taxonomic status of particular taxa (Ottenburghs 2019). The first criterion to consider is the level of reproductive isolation between taxa. If reproductive isolation is complete, the two taxa should be considered separate species. If reproductive isolation is incomplete, the level of genomic differentiation and diagnosability (e.g., differences in behavior or morphology) can be taken into account. Here, different scenarios are possible. For example, a high level of genomic differentiation in combination with several diagnostic features suggests a species status, whereas a low level of genomic differentiation in combination with no diagnostic features indicates that the taxa should be treated as subspecies. A special situation concerns the combination of low genomic differentiation and several diagnostic features. To reach a taxonomic decision, genomic islands of differentiation can be taken into account. If the diagnostic features can be linked to particular genomic islands of differentiation (thus providing a genetic basis for these features), the taxa can be considered distinct species. If not, a subspecies status is more appropriate.

**Fig. 5:**
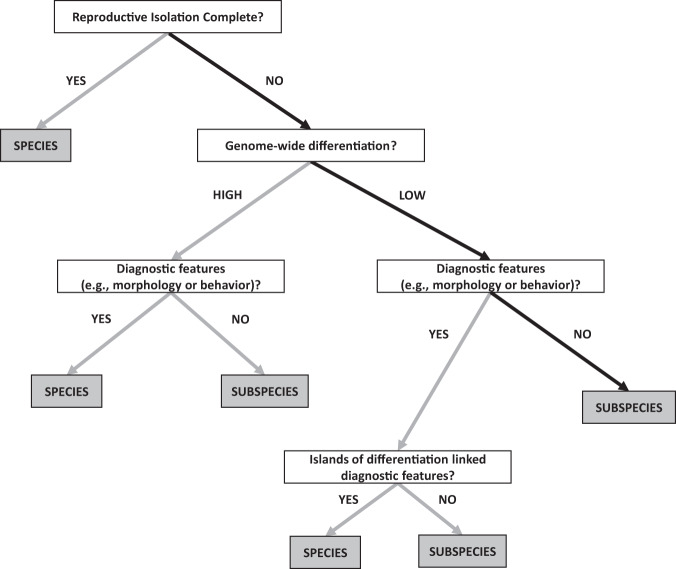
Decision tree for classification of species and subspecies based on reproductive isolation, genetic differentiation and morphology. The black arrows indicate the route followed to determine the taxonomic position of the Taiga and the Tundra Bean Goose.

To visualize this taxonomic decision process, we constructed a decision tree which we illustrate with the information on the Taiga and the Tundra Bean Goose (Fig. 5). First, reproductive isolation between the Taiga and the Tundra Bean Goose is incomplete: both taxa are known to hybridize (Ottenburghs et al. 2016a; Honka et al. 2017) and this study uncovered high levels of recent introgression. Second, although this study shows that they are genetically distinct, the degree of genetic differentiation is very low (genome-wide *F*_ST_ = 0.033). This level of genome-wide differentiation is lower compared to other bird systems that are considered subspecies, such as *Catharus* thrushes (*F*_ST_ = 0.1; Delmore et al. 2015) and some members of the Yellow-rumped Warbler (*Setophaga coronata*) complex (*F*_ST_ = 0.06; Irwin et al. 2018). One notable exception concerns the Golden-winged (*Vermivora chrysoptera*) and Blue-winged Warblers (*Vermivora cyanoptera*) that, despite a genome-wide *F*_ST_ of only 0.0045, are considered distinct species (Toews et al. 2016). Third, there are no clear diagnostic features to discriminate between the Taiga and the Tundra Bean Goose (de Jong 2019). Moreover, there is considerable morphological variation within both taxa (Burgers et al. 1991). Possibly, there is clinal variation in certain traits, such as beak size, across the range of the Bean Goose complex, similar to the Greater White-fronted Goose (*A. albifrons*, Ely et al. 2005). However, the morphology of the eastern Bean Goose taxa (*A. s. serrirostris* and *A. f. middendorfii*) will need to be assessed to obtain a complete picture of morphological variation within the Bean Goose complex. On the basis of the low genetic differentiation, considerable morphological variation and incomplete reproductive isolation, we argue that the Taiga and the Tundra Bean Goose should be treated as subspecies.

## Supplementary information

Supplementary Material

## Data Availability

The genome re-sequencing data are freely available in EMBL‐EBI European Nucleotide Archive (http://www.ebi.ac.uk/ena) under accession number PRJEB35788. The scripts and workflow for the analyses can be found on the following Github-page: https://github.com/JenteOttie/Goose_Genomics/tree/master/BeanGoose.
