# Supplementary material for: Recent introgression between Taiga Bean Goose and Tundra Bean Goose results in a largely homogeneous landscape of genetic differentiation
Source: Heredity (Edinb). 2020 May 26;125(1-2):73–84. doi: 10.1038/s41437-020-0322-z (PMC7413267; doi:10.1038/s41437-020-0322-z)
Supplement: Supplementary file 1 — Supplementary Material [file 41437_2020_322_MOESM1_ESM.pdf]

Supplementary Material

Table S1. Overview of sampling locations

Table S2. Mapping and coverage statistics for all samples.

Table S3. Correlations between F_{ST}, d_{XY} and π for different window sizes (10kb, 20kb, 50kb, 100kb and 200kb).

Table S4. Parameter estimates and likelihood scores for different demographic models simulated using DADI. The most likely model is highlighted in grey.

Table S5. Bootstrap analyses of the most likely demographic model. Parameters (with 95% confidence intervals) converted to absolute numbers.

Table S6. Gene content of the top 1% high F_{ST}-windows.

Figure S1. *ADMIXTURE* and PCA plots for different filtering settings.

Figure S2. *ADMIXTURE* plots for K = 2 to K = 4. The distribution of cross-validation errors indicates that K = 1 is the best configuration.

Figure S3. The genomic landscape of Taiga and Tundra Bean Goose for relative divergence (F_{ST}), absolute divergence (d_{XY}) and nucleotide diversity (π) aligned to chicken chromosomes. The colors in the nucleotide diversity tracks correspond to the Taiga (blue) and the Tundra Bean Goose (red).

Figure S4. Demographic scenarios with and without population expansions and their likelihood scores that were tested with DADI. The most likely model concerns isolation with recent asymmetrical gene flow without any population expansions (log likelihood = -31,804).

Table S1. Information about sampling locations for all goose specimens in this study. Species identity of Finnish samples based on mitochondrial DNA sequencing, other samples based on morphology.

SampleName	Code	Species	Collection	Location	Country
AnFa01U01	NRM 20006276	*A. fabalis*	Museum Stockholm	Kristianstad, Trolle-Ljungby	Sweden
AnFa01U02	NRM 20006268	*A. fabalis*	Museum Stockholm	NA	Sweden
AnFa01U03	20A2012	*A. fabalis*	J. Honka/University of Oulu	Lokka Tekoallas	Finland
AnFa01U04	25C2012	*A. fabalis*	J. Honka/University of Oulu	Liminka	Finland
AnFa01U05	112012	*A. fabalis*	J. Honka/University of Oulu	Selänpää	Finland
AnFa01U06	232012	*A. fabalis*	J. Honka/University of Oulu	Kankaanpää	Finland
AnFa01U07	242012	*A. fabalis*	J. Honka/University of Oulu	Pudasjärvi	Finland
AnFa01U08	33B2012	*A. fabalis*	J. Honka/University of Oulu	Vaala	Finland
AnFa01U09	32A2012	*A. fabalis*	J. Honka/University of Oulu	Toholampi	Finland
AnRo01F01	255-349	*A. serrirostris*	G. Müskens	Maren-Kessel	The Netherlands
AnRo01M01	255-346	*A. serrirostris*	G. Müskens	Maren-Kessel	The Netherlands
AnRo01U01	362012	*A. serrirostris*	J. Honka/University of Oulu	Kittilä	Finland
AnRo01U02	102012	*A. serrirostris*	J. Honka/University of Oulu	Rääkkylä	Finland
AnRo01U03	192012	*A. serrirostris*	J. Honka/University of Oulu	Hamina	Finland
AnRo01U04	14B2012	*A. serrirostris*	J. Honka/University of Oulu	Virolahti	Finland
AnRo01U05	382012	*A. serrirostris*	J. Honka/University of Oulu	Tyrnävä	Finland
AnRo01U06	22B2012	*A. serrirostris*	J. Honka/University of Oulu	Joutseno	Finland
AnRo01U07	412012	*A. serrirostris*	J. Honka/University of Oulu	Salo	Finland

Table S2. Mapping statistics for all samples.

Sample	Number of reads	Mapped reads	Mean Coverage
AnFa01U01	312,362,054	304,812,645 (97.58%)	38.30X
AnFa01U02	325,099,485	314,649,926 (96.79%)	39.58X
AnFa01U03	387,180,588	359,734,515 (92.91%)	40.05X
AnFa01U04	313,424,653	262,454,848 (83.74%)	30.73X
AnFa01U05	322,101,618	305,479,772 (94.84%)	37.62X
AnFa01U06	392,536,125	365,170,541 (93.03%)	42.99X
AnFa01U07	340,473,167	325,662,754 (95.65%)	38.84X
AnFa01U08	326,353,112	302,622,885 (92.73%)	36.00X
AnFa01U09	371,017,606	334,170,460 (90.07%)	38.41X
AnRo01F01	319,819,026	308,937,936 (96.6%)	38.90X
AnRo01M01	363,607,500	352,523,736 (96.95%)	43.93X
AnRo01U01	358,627,964	318,316,595 (88.76%)	35.36X
AnRo01U02	344,639,151	310,242,537 (90.02%)	34.87X
AnRo01U03	338,470,668	317,514,405 (93.81%)	36.84X
AnRo01U04	369,079,405	337,993,551 (91.58%)	39.45X
AnRo01U05	346,764,567	312,937,204 (90.24%)	32.71X
AnRo01U06	304,353,937	277,986,328 (91.34%)	33.57X
AnRo01U07	329,727,996	299,965,175 (90.97%)	34.74X

Table S3. Correlations between summary statistics (F_{st}, D_{xy} and π) for different window sizes.

**Correlation**	**Window Size**	**Spearman ρ**	**S-statistic**	**p-value**
Fst - Dxy	10kb	0.13	1.31e14	< 2.2e-16
	20kb	0.14	2.27e13	< 2.2e-16
	50kb	0.13	1.68e12	< 2.2e-16
	100kb	0.13	2.29e11	< 2.2e-16
	200kb	0.14	3.28e10	< 2.2e-16
Fst – Mean π	10kb	-0.044	1.58e14	< 2.2e-16
	20kb	-0.051	2.78e13	< 2.2e-16
	50kb	-0.088	2.11e12	< 2.2e-16
	100kb	-0.094	2.87e11	< 2.2e-16
	200kb	-0.088	4.13e10	< 2.2e-16
Dxy – Mean π	10kb	0.88	1.75e13	< 2.2e-16
	20kb	0.90	2.76e12	< 2.2e-16
	50kb	0.91	1.80e11	< 2.2e-16
	100kb	0.91	2.28e10	< 2.2e-16
	200kb	0.91	3.45e9	< 2.2e-16

Table S4. Parameter estimates and likelihood scores for different demographic models simulated using DADI.

**Model: Strict Isolation (no migration)**								
**Run**	**N1**	**N2**	**T**	**Likelihood**				
1	3.66	3.13	0.19	-472,672				
2	2.12	2.17	0.19	-501,858				
3	4.03	3.10	0.22	-554,411				
4	4.45	2.64	0.22	-560,482				
5	3.40	4.59	0.25	-628,042				
6	7.22	9.05	0.27	-733,355				
7	3.23	2.80	0.30	-737,060				
8	2.47	3.50	0.30	-750,695				
9	4.19	5.58	0.32	-777,150				
10	5.71	5.87	0.35	-837,773				
**Model: Isolation with continuous, symmetrical migration**								
**Run**	**N1**	**N2**	**T**	**m**	**Likelihood**			
1	0.86	0.82	7.65	8.10	-86,672			
2	1.03	1.00	9.83	6.63	-86,673			
3	0.67	0.65	5.54	9.95	-86,757			
4	1.05	1.01	9.13	7.23	-87,137			
5	1.27	1.20	9.44	5.36	-87,420			
6	1.00	0.96	6.65	7.17	-87,664			
7	0.63	0.69	4.80	9.55	-87,972			
8	0.92	0.88	4.89	7.31	-88,887			
9	0.56	0.59	4.61	9.76	-89,418			
10	1.02	0.85	9.63	9.56	-89,783			
**Model: Isolation with continuous, asymmetrical migration**								
**Run**	**N1**	**N2**	**T**	**m12**	**m21**	**Likelihood**		
1	0.57	0.35	12.91	12.12	20.54	-86,093		
2	0.68	0.45	10.97	10.37	15.64	-86,101		

3	0.87	0.61	12.08	8.45	11.29	-86,277		
4	0.61	0.33	15.67	9.96	20.82	-86,381		
5	1.39	1.06	14.74	5.39	6.71	-86,439		
6	0.58	0.65	7.96	12.36	10.15	-86,576		
7	0.69	0.79	10.01	11.41	9.16	-87,050		
8	0.86	0.40	16.88	6.27	18.52	-87,292		
9	0.56	1.06	12.42	14.18	5.11	-88,885		
10	0.76	0.62	11.02	6.77	9.55	-89,139		
**Model: Isolation with ancient, asymmetrical migration**								
**Run**	**N1**	**N2**	**Ts**	**Tm**	**m12**	**m21**	**Likelihood**	
1	0.63	0.54	20.77	4.81	10.77	13.10	-82,311	
2	0.81	0.91	8.46	5.85	9.10	7.27	-83,649	
3	0.67	0.95	12.80	5.26	11.89	7.64	-83,654	
4	0.85	0.62	5.36	14.23	7.73	11.09	-86,144	
5	1.41	1.30	15.45	18.94	5.11	5.30	-86,161	
6	1.48	1.42	17.11	19.45	4.86	4.97	-86,162	
7	1.35	1.18	19.33	25.71	5.26	6.03	-86,275	
8	0.90	0.84	9.59	18.49	7.73	8.39	-86,310	
9	1.09	1.11	15.20	23.40	6.54	6.02	-86,393	
10	0.90	0.50	19.18	18.69	9.85	15.72	-87,764	
**Model: Isolation with recent asymmetrical migration**								
**Run**	**N1**	**N2**	**Ts**	**Tm**	**m12**	**m21**	**Likelihood**	
1	1.36	0.68	14.36	0.33	2.89	14.61	-31,804	
2	1.25	0.52	12.50	0.32	2.32	13.82	-33,056	
3	1.52	0.51	14.53	0.32	2.46	12.44	-33,341	
4	1.62	0.64	13.73	0.38	2.53	14.71	-34,482	
5	1.53	0.66	9.07	0.30	2.62	13.20	-37,541	
6	1.74	0.72	9.88	0.35	2.31	11.08	-37,603	
7	1.88	0.70	12.93	0.47	2.26	11.88	-37,701	
8	1.19	0.77	13.98	0.37	2.15	12.95	-39,288	

9	1.40	0.69	7.44	0.30	2.90	13.08	-40,626	
10	1.52	0.86	11.04	0.43	2.20	10.84	-41,249	
**Model: Isolation with recent asymmetrical migration and population expansion (pop1)**								
**Run**	**s**	**N1**	**N2**	**Ts**	**Tm**	**m12**	**m21**	**Likelihood**
1	0.66	0.86	0.93	13.25	4.86	9.48	6.91	-83,236
2	0.42	0.70	0.70	19.03	4.46	16.23	8.05	-83,521
3	0.95	0.57	0.78	11.75	5.05	10.56	6.93	-83,721
4	0.70	0.81	1.11	9.81	4.26	10.04	4.54	-84,019
5	0.50	1.06	0.97	12.00	5.01	10.44	6.87	-84,024
6	0.30	1.23	0.93	17.03	4.70	17.15	6.70	-84,996
7	0.46	1.45	1.06	20.34	4.93	13.44	4.18	-85,494
8	0.48	0.70	0.84	17.05	9.52	13.15	7.89	-87,625
9	0.34	1.05	0.90	15.98	9.10	12.66	7.12	-89,796
10	0.42	0.60	0.68	15.07	3.45	18.74	5.04	-90,285
**Model: Isolation with recent asymmetrical migration and population expansion (pop2)**								
**Run**	**s**	**N1**	**N2**	**Ts**	**Tm**	**m12**	**m21**	**Likelihood**
1	0.59	1.00	0.50	16.15	5.46	6.33	11.37	-82,837
2	0.66	0.87	0.84	15	5.54	7.10	9.91	-82,866
3	0.73	1.25	0.58	10.86	4.82	4.25	10.50	-82,943
4	0.45	1.07	0.74	9.12	4.14	5.73	12.47	-82,952
5	0.44	0.97	0.64	21.51	5.88	5.72	15.42	-83,569
6	0.46	0.71	1.47	15.78	4.62	8.99	11.74	-83,631
7	0.79	1.12	0.79	12.84	5.70	7.51	8.12	-83,755
8	0.39	1.00	0.57	6.33	5.38	6.50	14.70	-86,103
9	0.60	1.23	0.59	15.86	4.88	5.35	11.42	-86,295
10	0.84	0.83	0.59	12.05	4.33	9.69	13.03	-86,528
**Model: Isolation with recent asymmetrical migration and population expansion (pop1&2)**								
**Run**	**s**	**N1**	**N2**	**Ts**	**Tm**	**m12**	**m21**	**Likelihood**
1	0.44	1.05	1.11	20.33	4.07	13.37	11.14	-83,672

2	0.58	1.79	0.67	11.98	3.56	9.91	16.38	-84,589
3	0.42	0.97	1.71	18.33	4.17	14.66	10.93	-85,346
4	0.36	1.02	0.74	14.39	3.63	22.02	9.95	-87,289
5	0.30	1.84	1.46	13.11	3.01	19.92	7.57	-87,572
6	0.54	0.98	0.45	16.96	7.50	9.01	15.34	-87,604
7	0.72	1.39	1.24	12.21	4.71	8.56	14.63	-88,112
8	0.25	0.65	0.45	9.75	8.76	11.95	11.80	-89,263
9	0.31	0.94	0.91	20.32	7.72	14.99	7.17	-89,619
10	0.65	0.75	0.78	10.39	7.43	9.32	10.10	-90,479

**Bootstrap Analyses**							
**Run**	**N1**	**N2**	**Ts**	**Tm**	**m12**	**m21**	**Likelihood**
1	1.36	0.70	15.90	0.32	2.77	14.29	-8.218
2	1.47	0.58	13.30	0.33	2.66	13.97	-8.253
3	1.26	0.56	15.46	0.35	2.21	14.54	-8.615
4	1.50	0.77	11.91	0.40	2.40	11.18	-10.245
5	1.30	0.57	14.72	0.22	2.88	14.72	-11.156
6	1.49	0.80	11.19	0.42	2.41	10.70	-11.312
7	1.07	0.53	15.20	0.22	2.70	15.87	-11.935
8	1.34	0.75	15.01	0.41	1.50	10.08	-12.676
9	1.15	0.65	15.96	0.22	2.78	14.14	-13.463
10	1.05	0.87	14.83	0.24	2.75	13.26	-14.807

**Final Results**			
	Coalescent Units (95 % CI)	***Absolute Units (95% CI)*** [1]	
**N (*A. fabalis*)**	1.3 (1.2 – 1.4)	120,508 (110,954 – 130,061) individuals	
**N (*A. serrirostris*)**	0.68 (0.61 – 0.75)	62,855 (56,102 – 69,608) individuals	
**Ts**	14.35 (13.32 – 15.38)	2,660,798 (2,470,104 – 2,851,492) years	
**Tm**	0.31 (0.26 – 0.37)	58,285 (48,651 – 67,918) years	
**m(F → S)**	2.51 (2.25 – 2.76)	0.0000121 (0.0000014 - 0.0000149)	
**m(S → F)**	13.27 (12.07 – 14.48)	0.0000651 (0.0000651 - 0.0000781)	

[1] Absolute units were calculated calculated using the following formulas and numbers:

Θ = 200 197 (theta. output from DADI-analyses)

μ = 1e10-9 (mutation rate)

L = 539 793 400 (length of sequence)

*N = Θ / (4 * μ * L)*

*N (A. fabalis) = N1 * N*

*N (A. serrirostris) = N2 * N*

*Ts = Ts * N*

*Tm = Tm * N*

m(S →F) = m12 / 2N

m(F →S) = m21 / 2N

Table S6. List of candidate genes in the top 1% F_{ST}-windows.

**Candidate Gene**	**Putative function (based on Uniprot)**
POLB	DNA repair
VDAC3	Channel for diffusion of small hydrophilic molecules
ANK1	Cytoskeleton organization
NKX6-3	Transcription factor (central nervous system?)
AGPAT6	Involved in several metabolic processes
KCNU1	Testis-specific potassium channel
UNC5D	Neuron differentiation
DUSP26	Phosphorylation of proteins
RNF122	May induce necrosis and apoptosis
TTI2	Regulator of the DNA damage response
MAK16	RNA-binding
SLC18A1	Neurotransmitter transport
ATP6V1B2	ATP-binding
LZTS1	Regulation of cell growth
GFRA2	Receptor for neurturin
PBDC1	Unknown function
GLI1	Transcriptional activator in development
STAC3	Muscle function

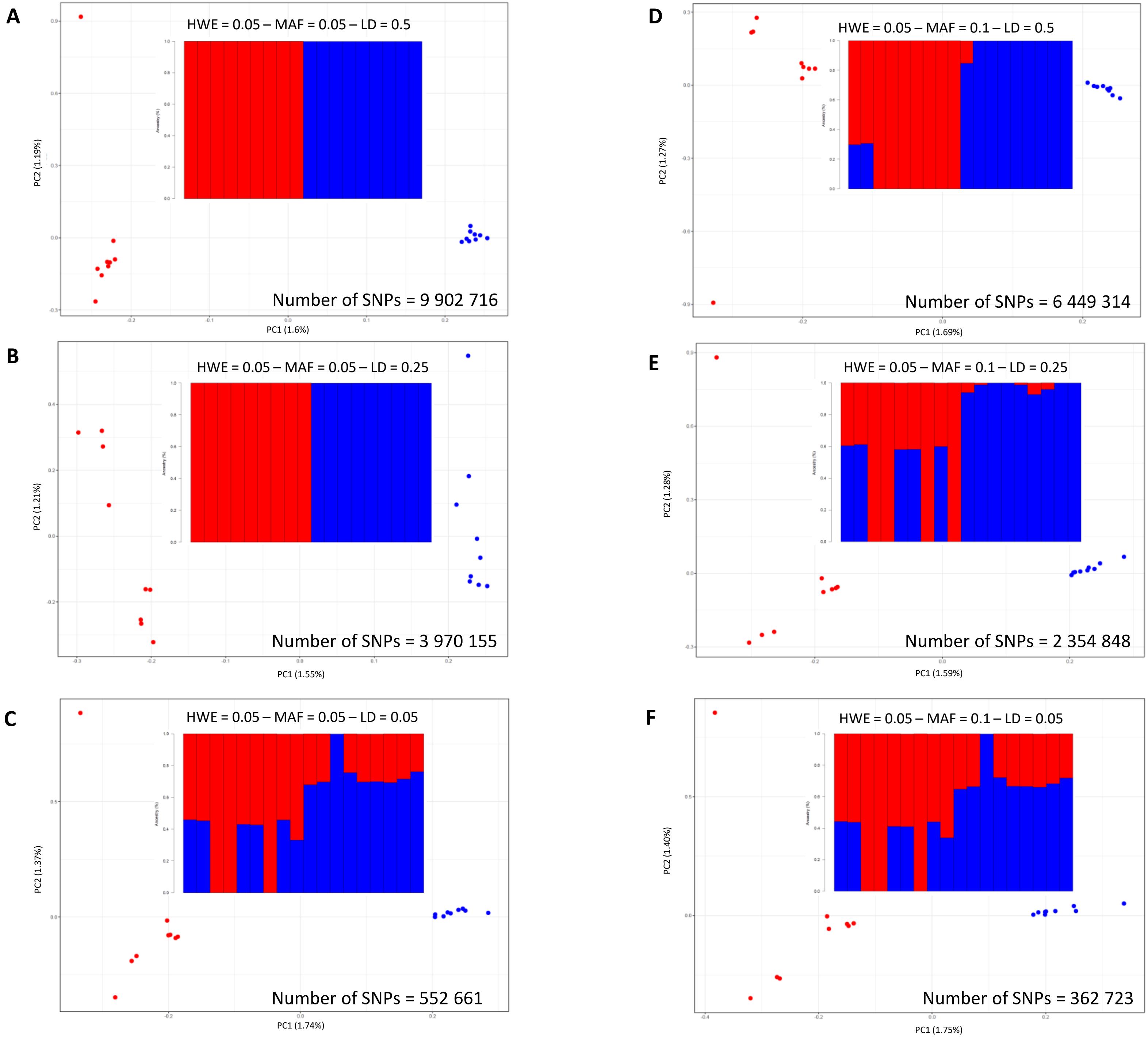
A
HWE = 0.05 – MAF = 0.05 – LD = 0.5
Number of SNPs = 9 902 716
PC1 (1.6%)
PC2 (1.19%)
B
HWE = 0.05 – MAF = 0.05 – LD = 0.25
Number of SNPs = 3 970 155
PC1 (1.55%)
PC2 (1.21%)
C
HWE = 0.05 – MAF = 0.05 – LD = 0.05
Number of SNPs = 552 661
PC1 (1.74%)
PC2 (1.37%)
D
HWE = 0.05 – MAF = 0.1 – LD = 0.5
Number of SNPs = 6 449 314
PC1 (1.69%)
PC2 (1.27%)
E
HWE = 0.05 – MAF = 0.1 – LD = 0.25
Number of SNPs = 2 354 848
PC1 (1.59%)
PC2 (1.28%)
F
HWE = 0.05 – MAF = 0.1 – LD = 0.05
Number of SNPs = 362 723
PC1 (1.75%)
PC2 (1.40%)

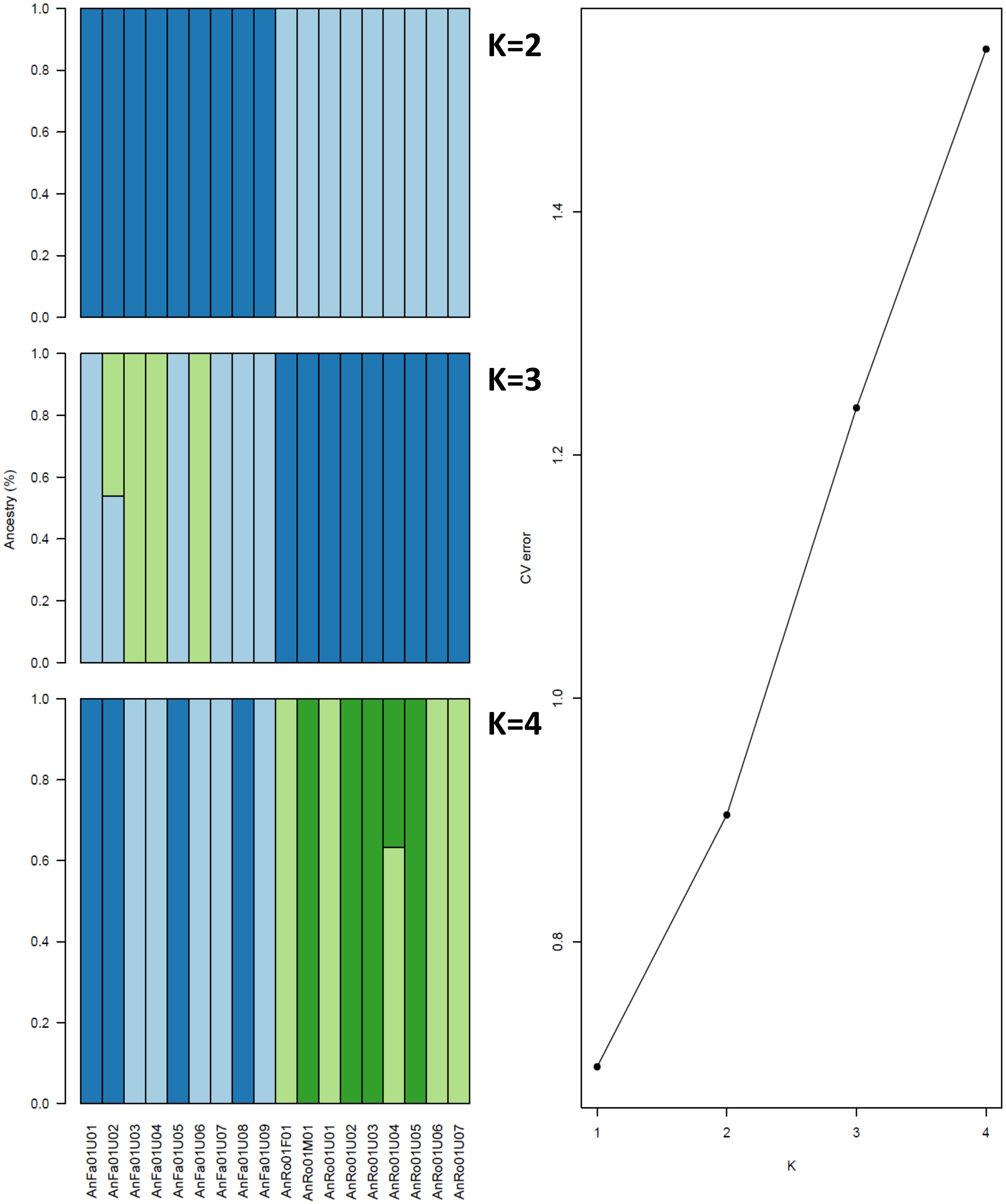
K=2
K=3
K=4
Ancestry (%)
CV error
K
AnFa01U01
AnFa01U02
AnFa01U03
AnFa01U04
AnFa01U05
AnFa01U06
AnFa01U07
AnFa01U08
AnFa01U09
AnRo01F01
AnRo01M01
AnRo01U01
AnRo01U02
AnRo01U03
AnRo01U04
AnRo01U05
AnRo01U06
AnRo01U07

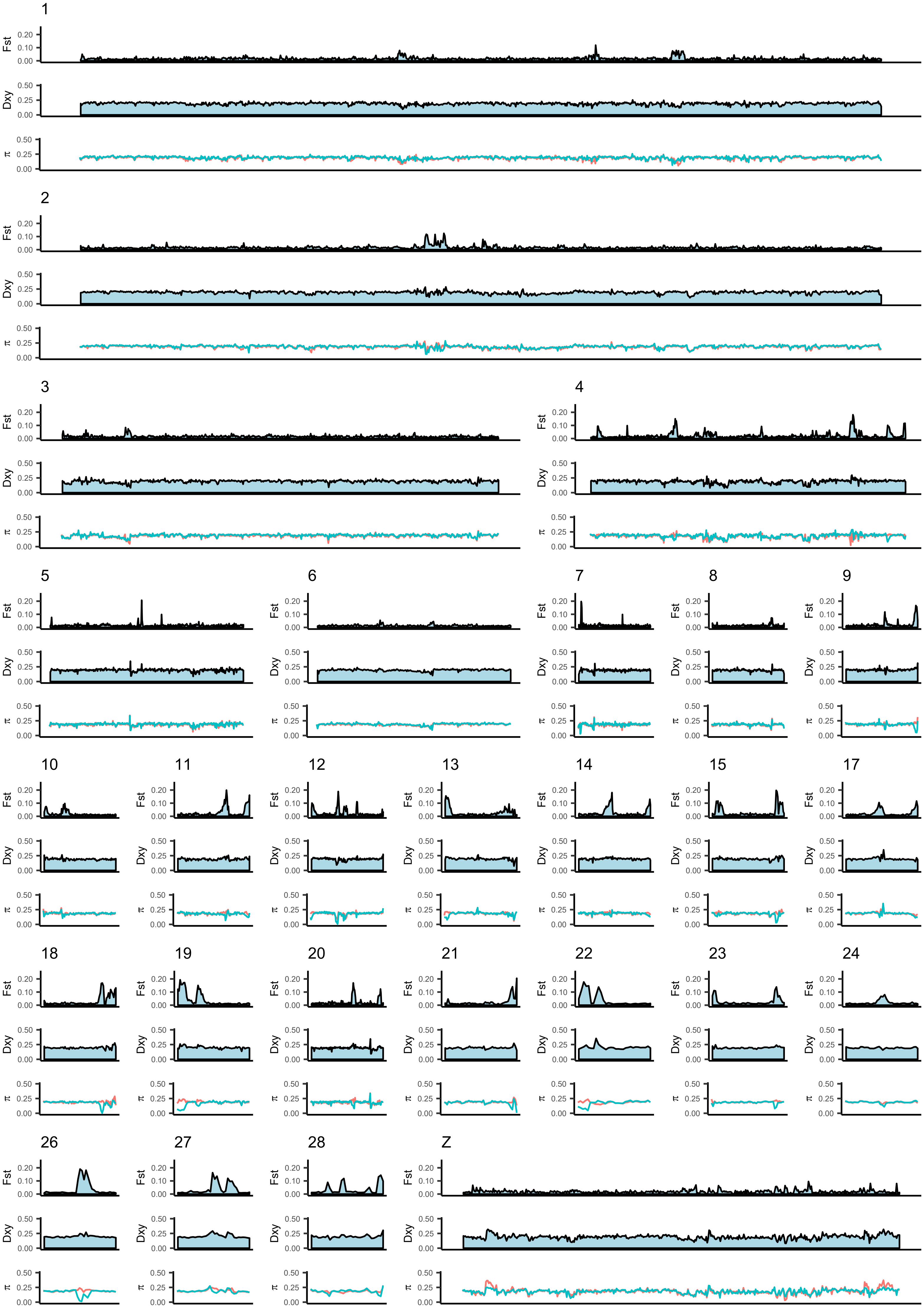

1
2
3
4
5
6
7
8
9
10
11
12
13
14
15
17
18
19
20
21
22
23
24
26
27
28
Z
Fst
Dxy
π
0.00
0.10
0.20
0.25
0.50

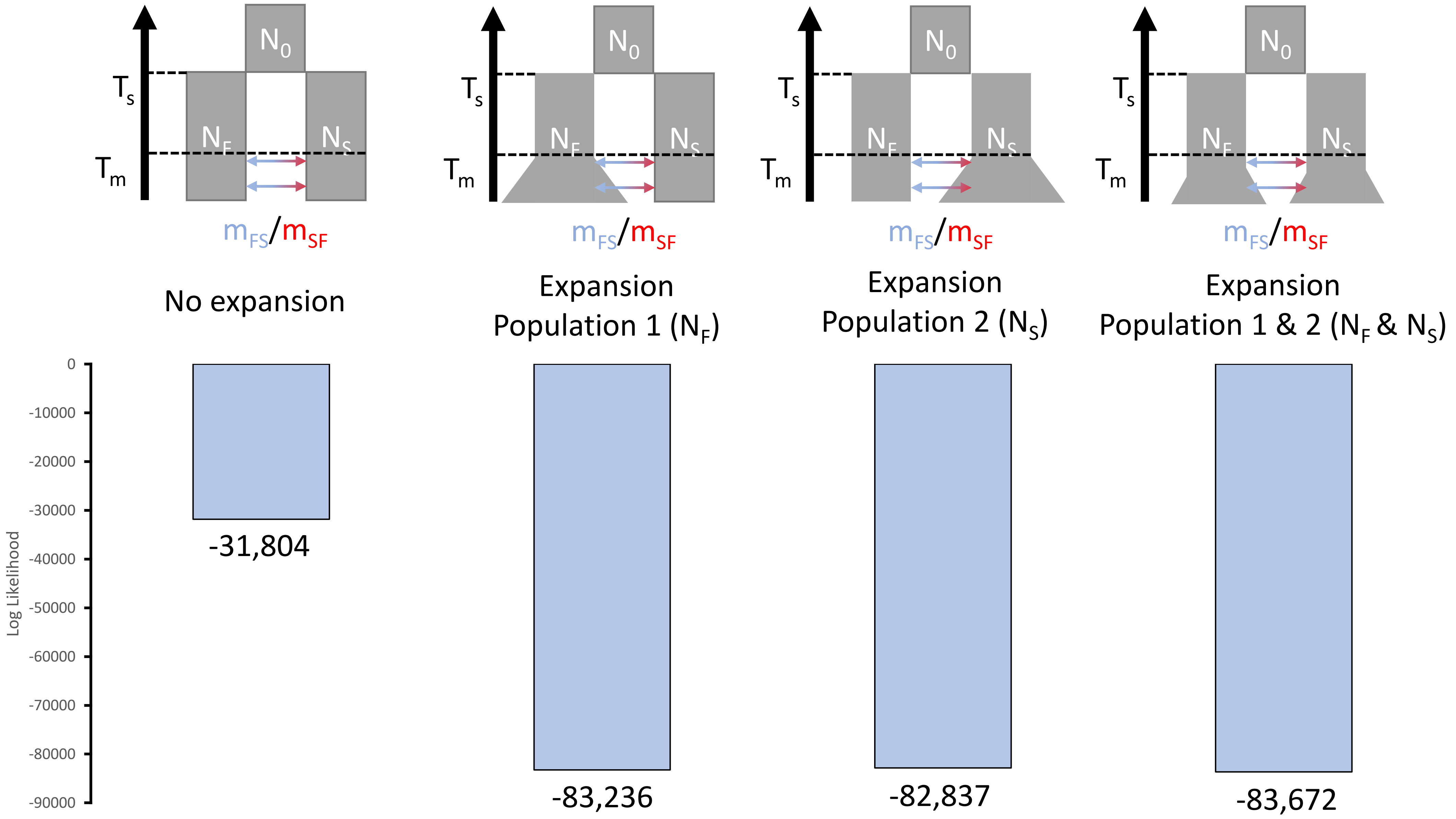

N0
Ts
NF
NS
Tm
mFS/mSF
No expansion
Expansion
Population 1 (NF)
Expansion
Population 2 (NS)
Expansion
Population 1 & 2 (NF & NS)
Log Likelihood
0
-10000
-20000
-30000
-40000
-50000
-60000
-70000
-80000
-90000
-31,804
-83,236
-82,837
-83,672
